# Probiotics in prevention and treatment of obesity: a critical view

**DOI:** 10.1186/s12986-016-0067-0

**Published:** 2016-02-20

**Authors:** Nazarii Kobyliak, Caterina Conte, Giovanni Cammarota, Andreana P. Haley, Igor Styriak, Ludovit Gaspar, Jozef Fusek, Luis Rodrigo, Peter Kruzliak

**Affiliations:** Division of Endocrinology and Metabolic Diseases, Catholic University of Sacred Heart, A. Gemelli Medical School, Rome, Italy; Division of Internal Medicine and Gastroenterology, Catholic University of Sacred Heart, A. Gemelli Medical School, Rome, Italy; Department of Psychology, The University of Texas at Austin, Austin, TX USA; University of Texas Imaging Research Center, Austin, TX USA; Institute of Geotechnics, Department of Biotechnology, Slovak Academy of Sciences, Kosice, Slovak Republic; 2nd Department of Internal Medicine, Comenius University and University Hospital, Mickiewiczova 13, 813 69 Bratislava, Slovak Republic; Faculty of Health Studies, University of Pardubice, Pardubice, Czech Republic; Department of Gastroenterology, Central University Hospital of Asturias (HUCA), Oviedo, Spain; 2nd Department of Internal Medicine, Faculty of Medicine, Masaryk University, Pekarska 53, 656 91 Brno, Czech Republic; Laboratory of Structural Biology and Proteomics, Central Laboratories, Faculty of Pharmacy, University of Veterinary and Pharmaceutical Sciences, Brno, Czech Republic

**Keywords:** Obesity, Prevention, Treatment, Gut microbiota, Intestinal microflora, Probiotics

## Abstract

The worldwide prevalence of obesity more than doubled between 1980 and 2014. The obesity pandemic is tightly linked to an increase in energy availability, sedentariness and greater control of ambient temperature that have paralleled the socioeconomic development of the past decades. The most frequent cause which leads to the obesity development is a dysbalance between energy intake and energy expenditure. The gut microbiota as an environmental factor which influence whole-body metabolism by affecting energy balance but also inflammation and gut barrier function, integrate peripheral and central food intake regulatory signals and thereby increase body weight. Probiotics have physiologic functions that contribute to the health of gut microbiota, can affect food intake and appetite, body weight and composition and metabolic functions through gastrointestinal pathways and modulation of the gut bacterial community.

## Background

Overweight and obesity are defined as abnormal or excessive fat accumulation that may impair health. Body mass index (BMI), defined as the weight in kilograms divided by the height in meters squared (kg/m2), is index that commonly used to classify overweight and obesity in adults due to its low cost and simplicity. The World Health Organization (WHO) [1] has defined overweight as having a BMI between 25.0 and 29.9 kg/m2; and obesity as having a BMI greater or equal than 30.0 kg/m2.

The worldwide prevalence of obesity more than doubled between 1980 and 2014 and for today WHO has declared obesity as global epidemic and took it under control. In 2014, more than 1.9 billion adults older than 18 years (39 %) are overweight. Overall, about 13 % or 600 million of these adult population (11 % of men and 15 % of women) were obese [1].

According to WHO the Global Database on BMI the prevalence of obesity is different globally and have sex-specific features. As of November 2004, the database include data from 350 separate surveys conducted over a 1942–2002 and has compiled results from a total of 97 countries and territories covering approximately 86 % of the adult population worldwide [2]. The highest prevalence of obesity is observed in the Pacific Islands and reach up to 80 % in some regions. The obesity rate less than 1 % has been reported in India [3].

In Europe, as for 2014, in general the incidence of obesity is high but geographically wide variations has been reported. The lowest rate observed in Tajikistan (13.5 %) and highest in Andorra and Turkey (29.4 %) [1].

More than 80 % of countries reported nationally gender-specific data related to prevalence of obesity or overweight in population. In majority of countries located in Africa, Latin America, Asia and Oceania the higher levels of overweight amongst women has been reported. In contrast to these regions in Europe and North America male’s overweight prevalence rates were more pronounced [2].

The National Health and Nutrition Examination Survey (NHANES) is another cross-sectional, nationally representative series of survey of 4115 adult men and women conducted in 1999 and 2000 by the National Center for Health Statistics of the U.S. Centers for Disease Control and Prevention. The age-adjusted prevalence of obesity and overweight in U.S. adults were 30.5 and 64.5 % respectively in 1999–2000 compared with 22.9 and 55.9 % in NHANES III (1988–1994; *P* < .001). Racial/ethnic groups did not differ significantly in the prevalence of obesity or overweight [4]. More recent data from NHANES show no significant changes in the prevalence of obesity for either men or women between 2003–2004 and 2005–2006 [5]. Interestingly that obesity prevalence was relatively low and stable between 1960 and 1980, but more than doubled from 15 % in 1980 to 34 % in 2006 [6] and keep the same rate and in nowadays – 33.4 % due to WHO-2014 data [1]. This possible stabilization in the obesity trends may be an early sign of a plateau in the obesity epidemic.

Children obesity is very important problem in whole word. In 2013, 42 million children under the age of 5 were overweight or obese. USA takes first place in the world for prevalence of obesity [1]. Childhood obesity has more than doubled in children and quadrupled in adolescents in the past 30 years [7]. The percentage of children aged 6–11 years in the U.S. who were obese increased from 7 % in 1980 to nearly 18 % in 2012. Similarly, the percentage of adolescents aged 12–19 years who were obese increased from 5 % to nearly 21 % over the same period [8]. In this country main factors contributing to the weight gain are widespread net of fast food restaurants and hypodynamic.

According to a new report published by the WHO Regional Office for Europe, the country profiles being overweight is so common that it risks becoming a new norm in the WHO European Region [9]. For example, up to 27 % of 13-year-olds and 33 % of 11-year olds are overweight. Among 11-year-old boys and girls, the prevalence of overweight was highest in Greece (33 %), Portugal (32 %), Ireland (30 %) and Spain (30 %) and lowest in the Netherlands (13 %) and Switzerland (11 %) [9].

The most frequent cause which leads to the obesity development is a dysbalance between energy intake and energy expenditure. In this complex process genetic susceptibility, environmental and lifestyle factors are involved. Current research efforts have focused on host and environmental factors that may affect energy balance.

Recent advances in next generation sequencing technology and mechanistic testing in gnotobiotic mice have identified the gut microbiota as an environmental factor which influence whole-body metabolism by affecting energy balance but also inflammation and gut barrier function, integrate peripheral and central food intake regulatory signals and thereby increase body weight. Underlying mechanisms whereby the gut microbiota contributes to host metabolism were revealed from studies on germ-free mice which were protected against developing diet-induced obesity [10, 11].

Consequently, the gut microbiota is gaining significant research interest in relation to obesity in an attempt to better understand the etiology of obesity and potentially new methods of its prevention and treatment [12].

## Pathophysiology of obesity and role of intestinal microflora in the metabolic steady state

The pathophysiology of obesity is multifactorial, and not completely understood. The obesity pandemic is tightly linked to an increase in energy availability, sedentariness and greater control of ambient temperature that have paralleled the socioeconomic development of the past decades [13]. Genetic factors also play a key role in determining body weight, with heritability estimates for the variance in body mass index ranging between 40 and 70 % [14, 15]. Epigenetic factors may also be involved [16]. For the purpose of this review, we will focus on the role of intestinal microflora in the pathophysiology of obesity and obesity-related metabolic alterations.

In recent years, intestinal microflora has received increasing attention as a metabolic gateway between the outer environment and the host, particularly with regard to modulation of inflammation, energy metabolism and body weight homeostasis. Human intestinal microflora represents a complex ecosystem consisting of trillions of microorganisms and thousands of bacterial species that are deeply involved in different functions of host metabolism [17]. A causal link between intestinal microflora and host metabolism was first provided when Turnbaugh et al. demonstrated that transplanting the intestinal microflora from obese mice could replicate the obese phenotype in germ-free mice [11]. Differences in intestinal microflora composition between humans seem to represent a key factor affecting energy homeostasis [18]. In both mice and humans, two bacterial phyla are dominant in the gut, the Gram-negative Bacteroidetes and the Gram-positive Firmicutes [18, 19]. Although few data exist at the genus and species level, studies in mice and in humans have demonstrated that obesity is associated with a reduction in the relative abundance of Bacteroidetes [18, 20, 21], and that the obese microflora has lower bacterial diversity than lean microflora [20, 22]. In overweight/obese humans, low faecal bacterial gene richness is associated with more marked overall adiposity and dyslipidaemia, impaired glucose homeostasis and higher low-grade inflammation [23, 24].

Intestinal microflora composition is strongly affected by dietary patterns. A high-fat and high-sugar Western diet increases the relative abundance of Firmicutes at the expense of the Bacteroidetes in animal models [22], whereas low-calorie diet induced weight loss may increase the relative abundance of Bacteroidetes in obese individuals [25]. Data obtained in animal models of the human intestinal ecosystem indicate that switching from a low-fat, plant polysaccharide-rich diet to a high-fat/high-sugar “Western” diet may shift the structure of the microflora within a single day, changing the representation of metabolic pathways and altering microbiome gene expression [26]. Consistently, a controlled-feeding study in humans showed that microflora composition changed detectably within 24 h of initiating a high-fat/low-fibre or low-fat/high-fibre diet, although the magnitude of the changes was modest [27]. Kong et al. explored differences in host inflammatory variables and intestinal microflora in function of three distinct dietary clusters in overweight/obese subjects. They found that subjects having a healthier dietary pattern (higher consumption of fruits, yogurt and soups and lower consumption of sweets, confectionary and table sugar and sugary drinks) showed less pronounced metabolic impairment and had the highest gene richness and diversity in their intestinal microflora, despite there was no difference in total energy intake or body weight across dietary clusters [28].

Several mechanisms by which the intestinal microflora may affect body weight have been proposed. The intestinal microflora of obese subjects may be more efficient at extracting energy from a given diet than the intestinal microflora of lean individuals [10, 11], which may lead to increased energy storage and adiposity. Another mechanism by which intestinal microflora may modulate energy intake and metabolism is the production of short chain fatty acids (SCFAs) from indigestible polysaccharides. SCFAs such as acetate, butyrate and propionate produced by bacterial fermentation function as energy substrates, as well as regulators of satiety and food intake [29]. By activating the G-protein-coupled receptors GPR41 and GPR43 on intestinal epithelial cells, SCFAs stimulate peptide YY (PYY) and glucagon-like peptide (GLP)-1 secretion. In turn, these hormones may suppress gut motility and retard intestinal transit, allowing for greater nutrient absorption [30, 31]. On the other hand, butyrate and propionate have been reported to reduce food intake and to protect against diet-induced obesity and insulin resistance in mice [32], greater bacterial gene richness has been associated with a higher production of SCFAs [24], and propionate-producer bacteria such as *Akkermansia muciniphila* may improve the metabolic profile in mice [33]. However, the role of SCFAs in the regulation of host metabolism has yet to be fully clarified. Finally, it has even been suggested that in Western societies the increased use of antibiotic treatment, i.e. one important factor that can affect microflora composition, may be associated with weight gain or obesity in humans [34].

A large body of evidence indicates that associations exist between alterations in intestinal microflora function and/or composition and metabolic derangements tightly linked to obesity, such as insulin resistance, atherosclerosis and low-grade chronic inflammation. Chronic low-grade inflammation appears to be a major factor in the development of obesity-related metabolic disturbances [35–37]. Studies in mice indicate that high-fat diet may result in changes in intestinal microflora composition and increased levels of circulating endotoxins such as lipopolysaccharide (LPS), and that infusion of LPS causes low-grade chronic inflammation and most of the features of the early onset of metabolic diseases, such as visceral fat deposition, glucose intolerance and hepatic insulin resistance [38]. LPS has been therefore postulated to be the molecular link between intestinal microflora and the chronic low-grade inflammation induced by a high-fat diet that leads to insulin resistance [39–41]. Abnormally increased gut permeability to bacteria and their products is a factor that further contributes to insulin resistance and oxidative stress [42, 43]. Consistently, observations in mice indicate that intestinal microflora influences energy metabolism and has systemic effects on host lipid metabolism, especially triglycerides and phosphatidylcholine [44]. The intestinal microflora has been shown to metabolize the dietary lipid phosphatidylcholine to trimethyl amine, which promotes atherosclerosis and inflammation in mice [45]. Of note, sequencing of the gut metagenome of patients with symptomatic atherosclerotic plaques revealed that symptomatic atherosclerosis is associated with an altered gut metagenome [46]. Finally, even tissue fatty acid composition may be modulated by intestinal microflora: beneficial intestinal Lactobacilli and Bifidobacteria can synthesize bioactive isomers of conjugated linoleic acid that have antidiabetic, anti-atherosclerotic, immunomodulatory, and anti-obesity properties [47].

Of note, the presence of obesity-related metabolic disturbances varies widely among obese individuals [48]. It would be interesting to explore whether differences in intestinal microflora have a role in determining the metabolically healthy and unhealthy obese phenotypes.

## Review of experimental studies

Preclinical evidence supporting the “anti-obesity” effects of probiotics mainly comes from studies on probiotics belonging to the genus Lactobacillus. Some other studies have focused on the use of Bifidobacterium strains.

Convincing evidence from animal studies suggests that probiotic administration may reduce, at least in part, the amount of weight gained in response to a high-fat diet (HFD). Probiotic supplementation with *Lactobacillus curvatus* HY7601 or *Lactobacillus curvatus* HY7601 in combination with *Lactobacillus plantarum* KY1032 effectively suppressed body weight gain and reduced the adipose tissue weight in mice fed a high-fat high-cholesterol diet for 9 weeks [49]. In another study by the same group, mice were fed a HFD for 8 weeks to induce obesity, and then randomized to receive HFD plus *Lactobacillus curvatus* HY7601 and *Lactobacillus plantarum* KY1032 or placebo for another 10 weeks. Mice fed normal chow served as controls. Following probiotic treatment, body weight gain was 38 % lower in the probiotic group than in the placebo group, although mice supplemented with probiotics still gained more weight than controls. Interestingly, the food efficiency ratio was reduced by a significant 29 % in mice supplemented with probiotics, indicating lower weight gain per grams of food consumed [50]. In keeping with these observations, it has recently been reported that 12-week dietary supplementation with either *Lactobacillus paracasei* CNCM I-4270, *Lactobacillus rhamnosus* I-3690 or *Bifidobacterium animalis subsp. lactis* I-2494 significantly attenuated HFD-induced weight gain despite no reductions in food intake in mice [51]. Similar results have been obtained in studies where *Bifidobacterium* spp. (*B. pseudocatenulatum* SPM 1204, *B. longum* SPM 1205, and *B. longum* SPM 1207 [52] or *B. adolescentis* [53]) was added to a HFD in rats.

Other probiotics shown to have anti-obesity effects include the plant-derived lactic acid bacterium *Pediococcus pentosaceus* LP28 [54], *Bacteroides uniformis* CECT 7771 [55] and *Akkermansia muciniphila* [33]. The latter is a mucin-degrading bacterium that resides and abundantly colonizes the mucus layer. The amount of these bacteria negatively correlates with body weight and decreases in response to a HFD [56]. Moreover, daily administration of *A. muciniphila* to HFD-induced obese mice for 4 weeks reduced body weight and improved body composition (i.e., fat mass/lean mass ratio), with no changes in food intake [33]. Finally the antiobesity properties of a probiotic yeast were examined for the first time in a recent study by Everard and coll [56]. *Saccharomyces boulardii Biocodex* administration reduced body weight gain and fat mass in obese and type 2 diabetic mice, and significantly changed the gut microbiota composition with an increased proportion of Bacteroidetes and a decreased amount of the phyla Firmicutes, Proteobacteria, and Tenericutes [56].

Importantly, preclinical evidence seems to indicate that the benefits on body weight translate into favourable metabolic effects, i.e. improvements in insulin resistance/glycemic control, amelioration of lipid profile and prevention of non-alcoholic fatty liver disease (NAFLD). Among others, the probiotic bacterial strain *Lactobacillus rhamnosus* GG (LGG) has been consistently shown to exert beneficial effects on glucose homeostasis. Treatment with LGG for 13 weeks during HFD in mice improved insulin sensitivity and reduced lipid accumulation by stimulating adiponectin secretion and consequent activation of AMPK, a key enzyme that controls cellular energy status [57]. Tabuchi and coll. showed that LGG decreased glycated hemoglobin and ameliorated glucose tolerance in streptozotocin-induced diabetic rats, possibly due to prevention of a decrease in insulin secretion [58]. More recently, it has been proposed that the anti-diabetic effect of LGG may be due to a reduction in endoplasmic reticulum stress and suppressed macrophage activation, which result in enhanced insulin sensitivity [59]. Several other probiotic strains have been tested as agents with potential antidiabetic effects. Administration of *Lactobacillus curvatus* HY7601 and *Lactobacillus plantarum* KY1032 prevented the development of high-fructose diet-induced metabolic syndrome, i.e. lowered plasma glucose, insulin and triglycerides levels. These effects were associated with a reduction in oxidative stress [50]. Studies using the traditional Indian yoghurt – dahi supplemented with probiotic strains of *Lactobacillus acidophilus* and *Lactobacillus casei* have shown that this product can improve the stigmata of diabetes, i.e. hyperglycemia and hyperinsulinemia, in high-fructose induced rat models of diabetes [60, 61]. Dietary supplementation with *Lactobacillus paracasei* CNCM I-4270, *Lactobacillus rhamnosus* I-3690 or *Bifidobacterium animalis subsp. lactis* I-2494 was shown to significantly attenuate HFD-induced hyperinsulinemia, hyperglycemia and glucose intolerance in mice [51]. *Bifidobacterium adolescentis* prevented insulin resistance in rats fed a HFD; interestingly, supplementation with this probiotic blunted the reduction in pancreas weight induced by the HFD, which may be related to the observed preservation of insulin sensitivity [53]. Finally, treatment with *A. muciniphila* for 4 weeks completely reversed diet-induced fasting hyperglycemia, possibly via a reduction in gluconeogenesis, and reduced insulin resistance in HFD-induced obese mice [33].

Probiotic supplementation also appears to be associated with a favorable effect on NAFLD, although different Lactobacillus and Bifidobacterium strains have specific effects on markers of obesity in rodent models. The analysis of more than 20 articles from 2013 to July 2014 by Cani and coll. showed that at least 15 different strains of Lactobacillus and three strains of Bifidobacterium do not equally influence on hepatic lipids and NAFLD development on different animal models. Remarkably, 12 strains decreased hepatic tissue inflammation and 11 reduced the hepatic triglyceride content when given as a single treatment [62].

Short-term courses of probiotic mixtures containing concentrated biomass of 14 alive probiotic strains (Lactobacillus, Lactococcus, Bifidobacterium, Propionibacterium, Acetobacter) from childhood was shown to reduce significantly total body and visceral adipose tissue weight, together with improvement in insulin sensitivity [63] and prevention of NAFLD development [64] in mice neonatally treated with monosodium glutamate. Similarly, administration of LGG protected mice from NAFLD development induced by a high-fructose diet through an increase of beneficial bacteria, restoration of gut barrier function and subsequent attenuation of liver inflammation and steatosis [65]. A recent study compared the effects of four Bifidobacteria strains (*Bifidobacteria* L66-5, L75-4, M13-4 and FS31-12) on lipid metabolism in high-fat diet obese mice. All four strains were associated with reductions in serum and liver triglycerides, and significantly alleviated lipid deposition in liver. Only *Bifidobacterium* L66-5 and *Bifidobacterium* FS31-12 decreased cholesterol liver content significantly [66]. Oral supplementation of *Bifidobacterium adolescentis* for 12 weeks was shown to protect C57BL/6 mice against diet-induced non-alcoholic steatohepatitis (NASH). Furthermore, mice treated with the probiotic had significantly decreased liver damage, which associated with prevention from lipid peroxidation, NFκB activation and finally inflammation in the liver [67]. Plaza-Diaz and coll. evaluated the effects of *Lactobacillus paracasei* CNCM I-4034, *Bifidobacterium breve* CNCM I-4035 and *Lactobacillus rhamnosus* CNCM I-4036 probiotic strains and their mixture on the development of hepatic steatosis as compared to placebo in Zucker rats with genetically determined obesity. In this study, only single-strain probiotic of *Lactobacillus rhamnosus* or *Bifidobacterium breve* and the mixture of *Bifidobacterium breve* and *Lactobacillus paracasei* decreased triacylglycerol content in liver and counteracted the development of hepatic steatosis in part by lowering serum LPS [68]. However, in another study, *Lactobacillus paracasei* CNCM I-4270, *Lactobacillus rhamnosus* I-3690 or *Bifidobacterium animalis subsp. lactis* I-2494 administered individually were all shown to protect against HFD-induced hepatic steatosis [51]. Finally, probiotics containing *Lactobacillus curvatus* HY7601 significantly reduced liver fat accumulation as compared to *Lactobacillus plantarum* KY1032 in diet-induced obesity. Combination of this probiotic was more effective for inhibiting gene expressions of various fatty acid synthesis enzymes in the liver, concomitant with decreases in fatty acid oxidation-related enzyme activities and their gene expressions [49]. Data from animal studies summarized in Table 1.

**Table 1:** Summary from animal studies of impact probiotic strains on obesity and associated diseases

Study	Experimental model of obesity	Type of probiotics	Duration of intervention	Key findings
Yoo et al. 2013 [49]	high-fat high-cholesterol diet (HFHCD)	*Lactobacillus curvatus* HY7601 alone or in combination with *Lactobacillus plantarum* KY1032	9 weeks parallel with HFHCD	↓ body weight gain ↓ hepatic lipid droplet accumulation and adipocyte size ↓ cholesterol in plasma and liver ↓ gene expressions fo fatty acid synthesis enzymes ↓ proinflammatory cytokines (TNF-α, IL-1b)
Park et al. 2013 [50]	HFD/**placebo or normal chow** for 8 weeks	*Lactobacillus curvatus* HY7601 and *Lactobacillus plantarum* KY1032	10 weeks after HFD	↓ body weight gain and fat accumulation ↓plasma insulin, leptin, total-cholesterol and liver toxicity biomarkers ↓ pro-inflammatory genes (TNFα, IL6, IL1β and MCP1) in adipose tissue ↓ fatty acid oxidation-related genes (PGC1α, CPT1, CPT2 and ACOX1)in the liver
Wang et al. 2015 [51]	HFD/**placebo or normal chow**	*Lactobacillus paracasei* CNCM I-4270, *Lactobacillus rhamnosus* I-3690 or *Bifidobacterium animalis subsp. lactis* I-2494	12 weeks parallel with HFD	↓ body weight gain ↓ macrophage infiltration into epididymal adipose tissue ↓ hepatic steatosis ↑ glucose–insulin homeostasis Strain-specific attenuation of obesity comorbidities throth impacts on MS-associated phylotypes of gut microbiota in mice
An et al. 2011 [52]	HFD/**normal chow**	Lactic acid bacterium (LAB) supplement (*B. pseudocatenulatum* SPM 1204, *B. longum* SPM 1205, and B*. longum* SPM 1207; 108 ~ 109 CFU)	7 weeks parallel with HFD	↓ body weight gain and fat accumulation ↓blood serum levels of total cholesterol, HDL-C, LDL-C, triglyceride, glucose, leptin ↓liver toxicity biomarkers (AST, ALT)
Chen et al. 2012 [53]	HFD/**normal chow**	*Bifidobacterium adolescentis*	12 weeks parallel with HFD	↓ body weight gain and visceral fat accumulation ↑insulin sensitivity
Zhao et al. 2012 [54]	HFD for 6 weeks/**normal chow**	*Pediococcus pentosaceus LP28 / Lactobacillus plantarum SN13T* as comparator	8 weeks after HFD	↓ body weight gain, visceral fat accumulation and liver lipid contents (triglyceride and cholesterol) ↓ hepatic lipid droplet accumulation and adipocyte size ↓ lipid metabolism-related genes (CD36, SCD1, PPARγ)
Gauffin et al. 2012 [55]	HFD/**normal chow**	*Bacteroides uniformis* CECT 7771	7 weeks parallel with HFD	↓body weight gain, visceral fat accumulation and liver lipid contents (triglyceride and cholesterol) ↑small adipocyte numbers ↓serum cholesterol, triglyceride, glucose, insulin and leptin levels, ↑oral tolerance to glucose ↓dietary fat absorption (reduced number of fat micelles in enterocytes) ↑ immune defence mechanisms
Everard et al. 2013 [33]	HFD/**normal chow**	*Akkermansia muciniphila(alive versus heat- killed)*	4 weeks parallel with HFD	↓body weight gain ↓metabolic endotoxemia and adipose tissue inflammation ↓insulin resistance ↑intestinal levels of endocannabinoids that control inflammation, the gut barrier, and gut peptide secretion - **all these effects required alive A. muciniphila because treatment with heat-killed cells did not improve the metabolic profile or the mucus layer thickness** - **administration of prebiotics (oligofructose) to ob/ob mice increased the abundance of A. muciniphila by ∼ 100-fold.**
	ob/ob mice/placebo (vehicle)			
Everard et al. 2014 [56]	db/db mice/**placebo (vehicle)**	*Saccharomyces boulardii Biocodex*	4 weeks	↓body weight gain and fat mass ↓hepatic steatosis and total liver lipids content ↓decreases hepatic (50 % decrease in CD11c macrophages level, F4/80, IL-1β and MCP-1mRNA) ↓systemic inflammation (↓plasma cytokine concentrations of IL-6, IL-4, IL-1β and TNF-α).
Kim et al. 2013 [57]	HFD/**normal chow**	*Lactobacillus rhamnosus GG*	13 weeks parallel with HFD	↓body weight gain and fat mass ↑insulin sensitivity, ↑expression of genes related to glucose metabolism (GLUT4 mRNA in skeletal muscle) ↑ adiponectin production in adipose tissue ↑ AMPK in skeletal muscle and adipose tissue
Tabuchi et al. 2003 [58]	Neonatally streptozotocin-induced diabetic rats/placebo (vehicle)	*Lactobacillus rhamnosus GG*	10 weeks	↓ HbA1c ↑oral tolerance to glucose
Park et al. 2015 [59]	db/db mice/placebo (vehicle)	*Lactobacillus rhamnosus GG*	4 weeks	↑ glucose tolerance ↑insulin-stimulated Akt phosphorylation and GLUT4 translocation in skeletal muscle ↓endoplasmic reticulum (ER) stress in skeletal muscle ↓M1-like macrophage activation in white adipose tissues ↑insulin sensitivity
Yadav et al. 2006 [60]	high-fructose diet/**normal chow**	*Lactococcus lactis*	42 days parallel with high-fructose diet	↓ HbA1c ↓fasting blood glucose, insulin, free fatty acids and triglyceride
Yadav et al. 2007 [61]	high-fructose diet/**normal chow**	*Lactobacillus casei/Lactobacillus acidophilus*	8 weeks parallel with high-fructose diet	↓ HbA1c ↓fasting blood glucose plasma insulin, total cholesterol, triacylglycerol, LDL-cholesterol, VLDL-cholesterol and blood free fatty acids ↓liver glycogen ↓thiobarbituric acid-reactive substances and ↑ reduced glutathione in liver and pancreatic tissues
Ritze et al. 2014 [65]	high-fructose diet/**without control**	*Lactobacillus rhamnosus GG*	8 weeks parallel with high-fructose diet	↓ liver inflammation and steatosis (protection from NAFLD development) ↓duodenal IκB protein levels and restoration of the duodenal tight junction protein concentration ↓ portal LPS ↓ TNF-α, IL-8R and IL-1β mRNA expression in the liver
Yin et al. 2010 [66]	HFD/**normal chow**	*Bifidobacteria L66-5, L75-4, M13-4 and FS31-12*	6 weeks parallel with HFD	↓liver triglyceride, total cholesterol and total lipid deposition (all 4 strains, but in strain-dependent manner, more pronounced for B. L66-5) ↓serum triglyceride and total cholesterol (all 4 strains, but in strain-dependent manner, more pronounced for B. L66-5 and B. FS31-12) ↓ body weight gain - *B. L66-5* ↑ body weight gain - *B. M13-4* No changes in body weight gain *L75-4 and FS31-12*
Reichold A et al. 2014 [67]	HFD/**normal chow**	*Bifidobacteria adolescentis*	12 weeks parallel with HFD	↓ body weight gain ↓liver inflammation and steatosis (protection from NASH development) ↓ formation of reactive oxygen species ↓ activation of NFκB No effect on portal LPS, TLR-4 and Myd-88 mRNA expression in livers
Plaza-Diaz et al. 2014 [68]	ob/ob mice/placebo (vehicle)	*Lactobacillus paracasei CNCM I-4034, Bifidobacterium breve CNCM I-4035 and Lactobacillus rhamnosus CNCM or mixture of 3 strains*	30 days	↓ triacylglycerol liver content (for L. rhamnosus, B. breve or the mixture) ↓ neutral lipids liver content (for all four probiotic groups) ↓ serum LPS levels (for all four probiotic groups) ↓ serum TNF-α levels (for B. breve, L. rhamnosus or the mixture) ↓ serum IL-6 levels (for L. paracasei)
Savcheniuk O et al. 2014 [63, 64]	Monosodium glutamate (MSG) induced obesity/placebo (vehicle)	14 alive probiotic strains (Lactobacillus, Lactococcus, Bifidobacterium, Propionibacterium, Acetobacter)	3 month	↓body weight gain and visceral fat accumulation ↓liver lipid contents (protection from NAFLD development) ↓serum cholesterol, triglyceride, glucose, insulin and leptin levels, ↑insulin sensitivity (decreased HOMA-IR, increased adipocitokine)

In summary, several preclinical studies have been conducted that used different bacterial strains, animal models and length of administration. Nearly all studies have shown some anti-obesity property of probiotics. Modulation of energy homeostasis (e.g. reduction in the food efficiency ratio) and anti-inflammatory/anti-oxidant effects appear to underlie the beneficial properties of probiotics. However, despite a large body of evidence supporting the anti-obesity and favourable metabolic effects of probiotics, it should be borne in mind that these effects may vary dramatically, depending both on the bacterial strain and on the host [69].

## Critical review of clinical studies

Prevention and management of obesity is proposed to begin in childhood when environmental factors exert a long-term effect on the risk for obesity in adulthood. Thus, identifying modifiable factors may help to reduce this risk. Therefore, the search of new non-toxic means of the obesity prevention is the urgent challenge of modern science. Recent evidence suggests that gut microbiota is involved in the control of body weight, energy homeostasis and inflammation and thus, plays a role in the pathophysiology of obesity. Prebiotics and probiotics are of interest because they have been shown to alter the composition of gut microbiota and to affect food intake and appetite, body weight and composition and metabolic functions through gastrointestinal pathways and modulation of the gut bacterial community [70, 71].

In recent multicenter, double-blind, randomized, placebo-controlled trial administration of fermented milk containing *Lactobacillus gasseri* SBT2055 for 12 weeks lead to significant reduction of abdominal visceral and subcutaneous fat areas an average of 4.6 and 3.3 % respectively as measured by computed tomography. Body weight and BMI also decreased significantly approximately on 1.5 % (*P* < 0.001) only in active treated group suggesting beneficial influence of Lactobacillus gasseri on metabolic disorders [72].

This study evaluate the impact of perinatal probiotic intervention on childhood growth patterns and the development of overweight during a 10-year follow-up. Administration of *Lactobacillus rhamnosus GG* started 4 weeks before expected delivery with extension for 6 months postnatally modify the growth pattern of the child by restraining excessive weight gain during the first years of life. The most pronounced changes were observed at the age of 4 years (*P* = 0.063), with lack of efficiency in the later stages of development [73].

In this systematic review, which include 5 human trial reports, were analyzed the role of probiotics, basically containing Lactobacillus sub-strains, in metabolic parameters modulation in patients with type 2 diabetes. The significant reduction of at list one of the primary outcome endpoints which include levels of fasting plasma glucose, postprandial blood glucose, HbA1c, insulin, insulin resistance and onset of diabetes were demonstrated in all studies. Regarding secondary outcome measures, i.e. lipid profiles, pro-inflammatory and anti-oxidant factors, only one human study failed to show any significant changes in any of these parameters [74]. Furthermore, in obese patients with metabolic syndrome, improvement of insulin sensitivity (median rate of glucose disappearance changed from 26.2 to 45.3 μmol/kg/min; *P* < .05) were demonstrated after small intestinal microbiota translocation from lean donors [75]. In opposite to this findings, Mazloom et al. also demonstrated that 6 weeks of oral treatment with probiotics contained *L. acidophilus, L. bulgaricus, L. bifidum* and *L. casei* decreased the concentration of triglycerides, IL-6 level and insulin resistance in type 2 diabetic patients; however the change were not significant [76].

Several clinical trials revealed promising effects of probiotics in improving liver function, fat metabolism and insulin resistance in patients with obesity related non-alcoholic fatty liver disease (NAFLD). First clinical evidence of beneficial effects of probiotics in patients with chronic liver disease comes from the study reported by Loguercio et al. in 2005 [77]. This trial comprised patients with NAFLD (*n* = 22), alcoholic fatty liver (*n* = 20), chronic hepatitis C (*n* = 20), and cirrhosis (*n* = 16). In patients with NAFLD administration of probiotic VLS#3 for 3 months showed improvement in ALT levels, as well as markers of lipid peroxidation, whereas significant changes in serum cytokines (TNF-alpha, IL-6, and IL-10) levels wasn’t observed as compared to patients with alcoholic fatty liver [77].

In present randomized, placebo-controlled trial conducted on 30 patients with histologically proven NAFLD a probiotic mixture containing 500 million of *Lactobacillus bulgaricus* and *Streptococcus thermophilus* per day during 3 months lead to significant decreased of liver aminotransferases activity only in active treated group. Anthropometric parameters and cardiovascular risk factors remained unchanged after treatment in both groups [78].

In this report Malaguarnera et al., studied if intervention of *Bifidobacterium longum* in combination with prebiotics containing fructo-oligosaccharides for 24 weeks are superior to lifestyle modification alone in the treatment of non-alcoholic steatohepatitis (NASH). Undoubted advantage of this study that liver biopsies were performed at entry and repeated for histological changes assessment in post-treatment period. In active treated group as compared to lifestyle modification alone significant reduction of TNF-α, CRP, serum AST levels, HOMA-IR and serum endotoxin. Furthermore, administration of *Bifidobacterium longum* in combination with prebiotics also lead to significant improvement of liver histology pattern. The mean NASH activity index decreased from 9.44 (range 6–10) at baseline to 3.22 (range 1–7) at 24 weeks [79].

In recent randomized clinical trial the treatment of patient with NASH for 6-month with the Lepicol probiotic formula contained *Lactobacillus plantarum, Lactobacillus deslbrueckii, Lactobacillus acidophilus, Lactobacillus rhamnosus* and *Bifidobacterium bifidum* lead to significant changes in intrahepatic triglyceride content, as measured by proton-magnetic resonance spectroscopy (22.6 ± 8.2 % to 14.9 ± 7.0 %; *P* = 0.034) as compared to usual care group (16.9 ± 6.1 % to 16.0 ± 6.6 %; *P* = 0.55). On the other hand, the use of probiotics was not associated with changes in BMI, waist circumference, glucose and lipid levels. Limitations of this study was the small sample size (*n* = 10, in each group) [80].

The use of probiotic may be beneficial in management of pediatric NAFLD. Recent study evaluated the effects of treatment with *Lactobacillus rhamnosus strain GG* (12 billion CFU/day) or placebo for 8 weeks in children with obesity-related NAFLD. After probiotic treatment were observed a significant decrease in aminotransferase activity. From the other hand, concentration of TNF-α and US bright liver parameters changes insignificantly and remained fairly stable [81].

The data from these four randomized trials involving 134 NAFLD/NASH patients were summarized in recent published meta-analysis [82]. The results showed that probiotic therapy significantly decreased aminotransferase activity, reduced total-cholesterol and TNF-α levels, parallel with improvement of insulin resistance. However, the use of probiotics was not associated with changes in BMI, glucose (GLU) and low density lipoprotein (LDL) (BMI: weighted mean difference (WMD) 0.05, 95 % CI: -0.18-0.29, *P* = 0.64; GLU: WMD 0.05, 95 % CI: -0.25-0.35, *P* = 0.76; LDL: WMD -0.38, 95 % CI: -0.78-0.02, P = 0.06).

Recent study found different therapeutic answer on probiotic administration in NAFLD patients depending on baseline aminotransferase activity [83]. Totally 72 patients with type 2 diabetes and NAFLD were included. Patients of active treated group received multistrain probiotic “Symbiter” containing concentrated biomass of 14 alive probiotic bacteria of *Bibidobacterium*, *Lactobacillus*, *Lactococcus*, *Propionibacterium* genera as adjunct to standard antidiabetic therapy. Statistically significant reduction of serum proinflammatory cytokines after 30 days of therapy were observed both in patients with normal or elevated baseline aminotransferase activity, but in elevated transaminases sub-group these changes more pronounced. In particular, the level of IL-6 decreased on 40 % (*p* = 0.041), IL-8 - 26.54 % (p <0.001), TNF-α - 20.83 % (*p* < 0.001), IL-1β - 17.7 % (*p* < 0.001) and interferon (IFN)-γ on 21.84 % (*p* < 0.001) respectively. In patients with normal levels transaminases and NAFLD were significantly decreased only IL-6 on 17.1 % (*p* = 0.041), IL-8 - 21.4 % (*p* < 0.001) and TNF-α on 13.8 % (*p* = 0.008). Patients of comparative group received only hypoglycemic drugs. Significant changes in serum cytokines levels in this were not observed.

Interesting findings were demonstrated in study reported by Shavakhi et al. were probiotic (Protexin two tablets per day) in combination with metformin improves liver aminotransferases better than metformin alone in patients with histologically proven NASH after 6 month of treatment. From the other hand lack of effectiveness changes for BMI, fasting glycemia, total-cholesterol and triglyceride were reported in both groups at the end of treatment [84].

Recent animal and clinical studies have revealed that probiotics may also improve liver injury in NAFLD. Alteration of gut-liver axis plays the pivotal role in the onset of fatty liver and related metabolic disturbances. Gut microbiota not only influences absorption and disposal of nutrients to the liver, but also can lead to the development of “metabolic endotoxemia” and activation of TLR ligands, which can stimulate liver cells to produce proinflammatory cytokines thereby initiate inflammation and fibrogenesis, which characterize NASH [85]. Another possible molecular mechanisms which implicated in NAFLD development are alteration in LPS- endocannabinoid (eCB) system regulatory loops and bile acids metabolism [62]. Thus, the modification of intestinal bacterial flora by specific probiotics has been proposed as a therapeutic approach for the treatment of NAFLD.

Several studies reported that dietary intervention with yoghurts containing probiotics (*Enterococcus faecium, Streptococcus thermophilus L. acidophilus*, *Bifidobacterium longum, L. plantarum* and/or *B. lactis*) significantly reduce total serum cholesterol and LDL cholesterol and to improve the LDL:HDL cholesterol ratio [86–88]. The present meta-analysis of controlled short-term intervention studies shows that the fermented yoghurt product produced a 4 % decrease in total cholesterol and a 5 % decrease in LDL-cholesterol when the open-label study is excluded. Different possible mechanism linking impact of probiotics on lipid metabolism [89]. These include the physiological action of the end-products of probiotic fermentation (acetate and propionate ratios), bacterial cholesterol assimilation, enzymatic deconjugation of bile acids, and cholesterol binding to the bacterial cell wall [90]. Some probiotic bacteria strains can affect blood cholesterol levels by expression of the bacterial enzyme bile salt hydrolase, which reduced readsorption of secreted bile acids, thereby driving the intestinal sink of cholesterol-derived bile acids and increasing bile acid synthesis in the liver and subsequent release in the intestine [91, 92]. Data from clinical studies are summarized in Table 2.

**Table 2:** Summary from clinical studies of impact probiotic strains on obesity and associated diseases

Study	Type of study	Type of probiotics	Duration of intervention	Key findings
Kadooka et al. 2010 [72]	multicenter, double-blind, randomized, placebo-controlled trial	*Lactobacillus gasseri SBT2055*	12 weeks	↓ abdominal visceral (on 4.6 %) and subcutaneous (3,3 %) fat areas as measured by CT ↓ body weight and BMI (on 1.5 %) ↑ serum adiponectin
Luoto et al. 2013 [50]	randomized, double-blind, prospective follow-up study	*Lactobacillus rhamnosus GG*	Mothers 4 weeks before expected delivery with extension for 6 months postnatally (in children)	↓ weight gain during the first years of life - most pronounced changes were observed at the age of 4 years (*P* = 0.063), with lack of efficiency in the later stages of development
Vrieze et al. 2012 [75]	randomized, double-blind, parallel, placebo-controlled trial (FATLOSE trial)	*allogenic microbiota from lean donors to male recipients with metabolic syndrome or autologous microbiota*	12 weeks	↑ insulin sensitivity (6 weeks after infusion) ↑levels of butyrate-producing intestinal microbiota.
Mazloom et al. 2013 [76]	randomized, single- blinded, placebo-controlled trial	*Lactobacillus* acidophilus, L. bulgaricus, L. bifidum, and L. casei	6 weeks	↓ triglyceride ↓ malondialdehyde (MDA) ↓IL-6 and ↓insulin resistance All changes not statistically significant
Aller et al. 2011 [78]	randomized, double-blind, parallel, placebo-controlled trial	*Lactobacillus bulgaricus vs Streptococcus thermophilus*	3 month	↓ liver aminotransferases levels in patients with NAFLD Anthropometric parameters and cardiovascular risk factors remained unchanged
Malaguarnera et al. 2012 [79]	open label study in patients with NASH	*Bifidobacterium longum* in combination with fructo-oligosaccharides (Fos) *versus* life style modification	24 weeks	Bifidobacterium longum with Fos when compared to lifestyle modification alone, significantly reduces TNF-α, CRP, serum AST levels, HOMA-IR, serum endotoxin, steatosis, and the NASH activity index.
Wong et al. 2013 [80]	randomized, open label study in patients with histology-proven NASH	Lepicol probiotic formula vs usual care	6 month	↓ intrahepatic triglyceride content (IHTG) ↓ AST level No changes in BMI, waist circumference, glucose and lipid levels
Vajro et al. 2011 [81]	double-blind, placebo-controlled pilot study in pediatric NAFLD	*Lactobacillus rhamnosus strain GG*	8 weeks	↓ALT TNF-α and US bright liver parameters remained fairly stable
Mykhalchyshyn et al. 2013 [83]	open label study in patients with NAFLD	“Symbiter” containing concentrated biomass of 14 alive probiotic bacteria	4 weeks	↓ IL-6, IL-8, TNF-α, IL-1β, IFN-γ (in elevated transaminases sub-group) ↓ IL-6, IL-8, TNF-α, (in normal transaminases sub-group)
Shavakhi et al. 2013 [84]	randomized, double-blind, placebo-controlled trial in patients with histology-proven NASH	probiotic Protexin plus Metformin 500 mg (Met/Pro) *versus* Metformin 500 mg plus placebo(Met/P)	6 month	Probiotic combination with Metformin improves liver aminotransferases better than metformin alone. BMI, fasting blood glucose, cholesterol, and triglyceride fell significantly in both groups.

## Conclusion

Lifestyle modifications still remain the primary therapy for obesity and the related metabolic disorders. Most of medications for treatment of obesity are taken out the production because of their adverse effects. Novel therapies targeting one or more of the underlying etiological factors are desirable. One of the potential ideal strategy for obesity treatment may be manipulation with gut microbiota. Firstly, this therapy is safe, due to absence of reported adverse effects, well-tolerated and appropriate for long-term use. Secondary, modulation of gut microbiota by probiotic treatment or dietary intervention due to it beneficial effects can affect body weight, influence on glucose and fat metabolism, improve insulin sensitivity and reduce chronic systemic inflammation. However, most prominent effect of probiotic on host metabolism from human studies are reported basically for *Lactobacillus* and/or *Bifidobacterium* strains. The general limitations of all this trials were small sample sizes and absence of longer-term follow up. Moreover currently, several potential bacterial candidates, such as *Saccharomyces cerevisiae var. boulardii, Enterobacter halii* or *Akkermansia muciniphila* have been identified and novel mechanisms of action governing their beneficial effects for obesity have been elucidated [62]. Taking into account all these data the concept of manipulating the gut microbiota to improve host metabolism has gained considerable interest nowadays. More evidence from human trials now needed to confirm beneficial effects of traditional probiotics for obesity and to conduct a meta-analysis. Another important research problem that arose for today became the search for next generation of probiotics for managing of obesity and its related disorders.
